# Ruxolitinib/nilotinib cotreatment inhibits leukemia-propagating cells in Philadelphia chromosome-positive ALL

**DOI:** 10.1186/s12967-017-1286-5

**Published:** 2017-08-30

**Authors:** Yuan Kong, Yi-Lin Wu, Yang Song, Min-Min Shi, Xie-Na Cao, Hong-Yan Zhao, Ya-Zhen Qin, Yue-Yun Lai, Hao Jiang, Qian Jiang, Xiao-Jun Huang

**Affiliations:** 1 Peking University People’s Hospital, Peking University Institute of Hematology, Beijing Key Laboratory of Hematopoietic Stem Cell Transplantation, Collaborative Innovation Center of Hematology, Peking University, Beijing, 100044 China; 2 Peking-Tsinghua Center for Life Sciences, Academy for Advanced Interdisciplinary Studies, Peking University, Beijing, China

**Keywords:** Acute lymphoblastic leukemia, Ph-chromosome, Leukemia-propagating cells, Nilotinib, Ruxolitinib

## Abstract

**Background:**

As one of the major treatment obstacles in Philadelphia chromosome-positive acute lymphoblastic leukemia (Ph^+^ALL), relapse of Ph^+^ALL may result from the persistence of leukemia-propagating cells (LPCs). Research using a xenograft mouse assay recently determined that LPCs were enriched in the CD34^+^CD38^−^CD58^−^ fraction in human Ph^+^ALL. Additionally, a cohort study demonstrated that Ph^+^ALL patients with a LPCs phenotype at diagnosis exhibited a significantly higher cumulative incidence of relapse than those with the other cell phenotypes even with uniform front-line imatinib-based therapy pre- and post-allotransplant, thus highlighting the need for novel LPCs-based therapeutic strategies.

**Methods:**

RNA sequencing (RNA-Seq) and real-time quantitative polymerase chain reaction (qRT-PCR) were performed to analyze the gene expression profiles of the sorted LPCs and other cell fractions from patients with de novo Ph^+^ALL. In order to assess the effects of the selective BCR–ABL and/or Janus kinase (JAK)2 inhibition therapy by the treatment with single agents or a combination of ruxolitinib and imatinib or nilotinib on Ph^+^ALL LPCs, drug-induced apoptosis of LPCs was investigated in vitro, as well as in vivo using sublethally irradiated and anti-CD122-conditioned NOD/SCID xenograft mouse assay. Moreover, western blot analyses were performed on the bone marrow cells harvested from the different groups of recipient mice.

**Results:**

RNA-Seq and qRT-PCR demonstrated that JAK2 was more highly expressed in the sorted LPCs than in the other cell fractions in de novo Ph^+^ALL patients. Combination treatment with a selective JAK1/JAK2 inhibitor (ruxolitinib) and nilotinib more effectively eliminated LPCs than either therapy alone or both in vitro and in humanized Ph^+^ALL mice by reducing phospho-CrKL and phospho-JAK2 activities at the molecular level.

**Conclusions:**

In summary, this pre-clinical study provides a scientific rationale for simultaneously targeting BCR–ABL and JAK2 activities as a promising anti-LPCs therapeutic approach for patients with de novo Ph^+^ALL.

## Background

The Philadelphia chromosome (Ph), the result of a balanced translocation between chromosomes 9 and 22, leads to the constitutively active breakpoint cluster region, Abelson (BCR–ABL) tyrosine kinase, which is critical for the pathogenesis of both chronic myeloid leukemia (CML) and Ph-positive acute lymphoblastic leukemia (Ph^+^ALL) [1–3]. With the widespread use of BCR–ABL tyrosine kinase inhibitors (TKIs), the prognosis of Ph^+^ALL has improved, but most patients relapse [4–6]. Some patients with Ph^+^ALL develop resistance to TKIs [7–9] but others may relapse due to the persistence of quiescent leukemia-propagating cells (LPCs) [10–12] which are defined by their capacity to initiate human leukemia and to self-renew in immunocompromised mice [13–17]. High-dose chemotherapy, TKIs, and even lethal conditioning before allogeneic hematopoietic stem cell transplantation (allo-HSCT) kill leukemia cells but cannot effectively eliminate LPCs.

Using an anti-CD122-conditioned non-obese diabetic/severe combined immunodeficiency (NOD/SCID) xenograft mouse assay, we previously reported that LPCs were enriched in the CD34^+^CD38^−^CD58^−^ fraction in human Ph^+^ALL [10]. Furthermore, a cohort study demonstrated that Ph^+^ALL patients with LPCs phenotype at diagnosis exhibited a significantly higher cumulative incidence of relapse than did the group with other cell phenotypes, even when receiving uniform front-line imatinib-based therapy pre- and post-allotransplant [18]. Therefore, it is imperative to identify novel therapeutic targets based on LPCs to improve the prognosis of Ph^+^ALL patients.

The Janus kinase (JAK)2-signal transducer and activator of transcription (STAT) pathway, which contains central components of hematopoietic cytokine receptor signaling pathways, participates in vital cellular functions, such as supporting the survival and proliferation of leukemia cells in the bone marrow (BM) microenvironment [19–22].

Recent evidence has demonstrated that CML stem/progenitor cells are able to survive independent of BCR–ABL kinase activity, suggesting that other pathways contribute to the persistence of these cells [23–25]. Among these, JAK2 is a component of the BCR–ABL network pathway and is activated in CML stem/progenitor cells [26–28]. Ruxolitinib was the first-in-class JAK1/JAK2 inhibitor approved for the treatment of primary myelofibrosis [29–31]. Gallipoli et al. [28]. reported that the combination of ruxolitinib and nilotinib, a second-generation TKI that is more potent than imatinib in CML, resulted in the enhanced eradication of CML stem/progenitor cells, highlighting JAK2 as a novel therapeutic target in CML stem/progenitor cells.

De novo Ph^+^ALL closely resembles the aggressive lymphoid blast crisis of CML and is prone to relapse even after combined treatment with potent second-generation TKIs and allo-HSCT. Abundant evidence suggests that JAK2 is an ideal target for anti-CML stem/progenitor cell therapy [26–28]. Moreover, Bi et al. reported that increased Th17 cells and IL-17A existed in patients with B cell acute lymphoblastic leukemia (B-ALL) and promoted the proliferation of B-ALL cells through activation of PI3K/Akt and JAK2/STAT3 signaling [32]. However, the JAK2 expression pattern in LPCs and the therapeutic effect of JAK2 inhibitor to eradicate LPCs in Ph^+^ALL remain to be explored.

In the current study, we aimed to compare the gene expression profiles between the sorted LPCs and other cell fractions from patients with de novo Ph^+^ALL. Moreover, we aimed to investigate whether selective BCR–ABL/JAK2 dual inhibition therapy using nilotinib combined with ruxolitinib could more effectively eliminate imatinib-insensitive LPCs in vitro and in humanized Ph^+^ALL mice and the underlying molecular mechanisms of this therapy.

## Methods

### Patients

Six patients with de novo Ph^+^ALL diagnosed at Peking University Institute of Hematology from January 1, 2015 to May 31, 2015 were enrolled for this in vitro and in vivo study. The patient clinical characteristics are shown in Table 1. The inclusion criteria were (1) 18–60 years of age, (2) a diagnosis of ALL based on the 2008 World Health Organization (WHO) criteria, and (3) the detection of the Ph-chromosome and/or *BCR–ABL* mRNA. Bone marrow mononuclear cells (BMMNCs) from the patients at diagnosis were rapidly isolated by density centrifugation using a lymphocyte separation medium (GE Healthcare, Milwaukee, WI, USA). The BMMNCs were immediately cryopreserved in 10% dimethyl sulfoxide (Sigma, St. Louis, MO, USA) with 90% fetal bovine serum (FBS, Gibco, Gaithersburg, MD, USA). The BMMNCs were stored in liquid nitrogen until the cell sorting procedure was performed. The study was approved by the Ethics Committee of Peking University People’s Hospital, and written informed consent was obtained from all patients before study-entry in accordance with the Declaration of Helsinki.

**Table 1:** Clinical characteristics of the patients with de novo Ph^+^ALL for in vitro and in vivo study

Case no.	Age/sex	WBC (×10E + 9/L)	% CD34^+^ in blasts	% CD34^+^CD38^−^ CD58^−^ in blasts	*BCR–ABL* (P190/P210)	Cytogenetics	Outcome
1	F/23	26.3	90.5	5.6	P210	46, XX, t(9;22) [20]	CCR
2	M/46	86.5	82.3	12.2	P210	46, XY, t(9;22) [20]	Relapsed
3	M/40	65.3	86.5	8.6	P190	46, XY, t(9;22) [20]	Relapsed
4	M/32	56.6	78.5	10.2	P190	46, XY, t(9;22) [20]	CCR
5	F/50	167.3	98.6	11.6	P190	46, XX, t(9;22) [20]	Relapsed
6	M/22	78.2	92.3	9.8	P190	46, XY, t(9;22) [20]	Relapsed

F, female; M, male; CCR, continuous complete remission

### Cell sorting of the LPCs and other cell fractions in the Ph^+^ALL patients

**Fig. 1:**
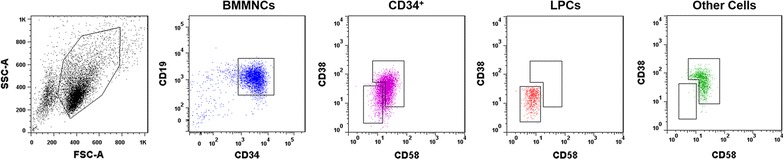
Representative flow cytometric analysis of a Ph^+^ALL patient sample sorted according to the distribution of CD34, CD38 and CD58 expression. In the viable bone marrow mononuclear cells (BMMNCs) of a de novo Ph^+^ALL patient, the LPCs (CD34^+^CD38^−^CD58^−^) and other cells (CD34^+^CD38^−^CD58^+^, CD34^+^CD38^+^CD58^−^ and CD34^+^CD38^+^CD58^+^) fractions were sorted simultaneously

The frozen BMMNCs of de novo Ph^+^ALL patients (N = 6) were thawed and stained with mouse anti-human CD58-FITC (Beckman-Coulter, Brea, CA, USA) and CD34-PE, CD19-APC-Cy7, CD45-PerCP, CD38-APC monoclonal antibodies (MoAbs, Becton–Dickinson, San Jose, CA, USA). The LPCs (CD34^+^CD38^−^CD58^−^) and other cell fractions (including CD34^+^CD38^−^CD58^+^, CD34^+^CD38^+^CD58^−^ and CD34^+^CD38^+^CD58^+^) in the viable BMMNCs were defined and sorted using a FACS Aria II (Becton–Dickinson) as previously reported^12^ (Fig. 1). The purity of each fraction was >97%. Fluorescence-minus-one controls were used to identify positive events for CD34, CD38 and CD58. The data were analyzed using BD LSRFortessa software (Becton–Dickinson).

### RNA sequencing (RNA-Seq), real-time quantitative polymerase chain reaction (qRT-PCR), and data analysis

To search for the potential molecular basis involved in LPC-mediated Ph^+^ALL progression, we performed RNA-Seq with the sorted LPCs and other cell fractions from the patients with de novo Ph^+^ALL (N = 2) to analyze their gene expression profiles. Total RNA was isolated from pellets using the RNeasy Mini Kit (Qiagen, Valencia, CA, USA). Three micrograms of RNA per sample was used as input material, and sequencing libraries were generated using the NEBNext Ultra RNA Library Prep Kit for Illumina (NEB, Ipswich, MA, USA). The library sequencing was performed on an Illumina HiSeq 2500 platform, and 125-bp paired-end reads were analyzed. Downstream analysis was performed using a combination of programs, including Bowtie2, Tophat2, HTseq, Cufflink and our wrapped scripts. The DESeq R package (1.10.1) was used to analyze the differential expression between the LPCs and other cell fractions. In all statistical analyses, *P*-values were analyzed using the Benjamini-corrected modified Fisher’s exact test, and a *P* < 0.05 was considered statistically significant.

To confirm the RNA-Seq results, the relative *JAK2* mRNA levels (forward primer: 5′-TCTGGGGAGTATGTTGCAGAA-3′; reverse primer: 5′-AGACATGGTTGGGTGGATACC-3′) between the LPCs and other cell fractions sorted from the patients with de novo Ph^+^ALL (N = 6) were analyzed using a SYBR green-based qRT-PCR technique. Normalized levels of the *JAK2* ratios in the qRT-PCR assays were evaluated through comparisons with the *GADPH* levels (forward primer: 5′- GCACCGTCAAGGCTGAGAAC -3′; reverse primer: 5′- TGGTGAAGACGCCAGTGGA -3′).

### Selective BCR–ABL and/or JAK2 inhibition for treating Ph^+^ALL LPCs in vitro

The sorted LPCs (1 × 10^5^/well) were cultured in StemSpan (Stem cell Technologies, Vancouver, BC, Canada) supplemented with four growth factors (20 ng/mL recombinant human (rh) interleukin-3 (rhIL-3), 20 ng/mL rhIL-7, 20 ng/mL rh Flt3-ligand (rhFlt3-L), and 50 ng/mL rh stem cell factor (rhSCF)) (PeproTech, Locky Hill, NJ, USA). After 48 h of suspension culture with the vehicle (DMSO, Sigma), imatinib (5 μM, Novartis, Basel, Switzerland) [24, 33], nilotinib (5 μM, Novartis) [33], and/or ruxolitinib (300 nM, Novartis) [34, 35] treatments, an apoptosis assay was performed on the LPCs using the Annexin-V and 7-amino-actinomycin D (7-AAD) Apoptosis Detection Kit (Becton–Dickinson) as described in our previous reports [36, 37]. The late apoptotic cells (Annexin-V^+^/7-AAD^+^) were analyzed using the BD LSRFortessa software (Becton–Dickinson). Aliquots of isotype-identical antibodies served as negative controls.

### Mice and xenograft assay

The anti-mouse CD122 [interleukin-2 receptor β (IL-2Rβ)]-conditioned NOD/SCID xenograft assay was performed with intra-bone marrow injection (IBMI) as previously reported [10, 38–40]. Briefly, 5- to 6-week-old NOD/SCID mice (Vital River Laboratories, Beijing, China) were sub-lethally irradiated (2.1 Gy total body irradiation from a ^60^Co source) followed by treatment with 200 μg of anti-mouse CD122 monoclonal antibody generated from hybridoma TM-β1 (provided by Dr. T. Tanaka of Hyogo University of Health Sciences, Kobe, Japan) [38]. The mice then received an IBMI of the LPCs fraction sorted from de novo Ph^+^ALL patients (N = 6) < 24 h post-irradiation and CD122 treatment. The injected LPCs doses were 1 × 10^4^ cells/mouse (6 mice for each treatment condition). All animal experiments were approved by the Ethics Committee of Peking University People’s Hospital.

### Selective BCR–ABL and/or JAK2 inhibition therapy in Ph^+^ALL LPCs-xenografted NOD/SCID mice

To evaluate the effects of selective BCR–ABL and/or JAK2 inhibition therapy on Ph^+^ALL LPCs, the recipient mice were treated with vehicle (10% 1-methyl-2-pyrrolidone and 90% polyethylene glycol 300; Sinopharm Chemical Reagent Co., Ltd, Shanghai, China) or with a single or different dual combination oral gavage treatments with imatinib (100 mg/kg/day, Novartis), nilotinib (75 mg/kg/day, Novartis), and/or ruxolitinib (30 mg/kg/day, Novartis) simultaneously once or twice a day starting at 2 weeks post-transplant and lasting for 2 weeks. The mice were observed daily for body weight changes and survival during and after treatment.

Engraftment of human Ph^+^ALL cells was defined based on the frequency of hCD45^+^ cells. Moribund mice were euthanized at 8 and 12 weeks post-transplant, and a mixture of cells from tibias, femurs and the spleen were obtained. To assess human Ph^+^ALL cell engraftment, flow cytometric analyses were performed using mouse anti-hCD58-FITC (Beckman-Coulter), CD34-PE, CD19-APC-Cy7, CD45-PerCP, and CD38-APC MoAbs (Becton–Dickinson).

To evaluate the engraftment levels of *BCR/ABL*-expressing cells, *BCR/ABL* transcripts were detected in BM cells from murine recipients using a TaqMan-based qRT-PCR assay performed as previously described [10]. *BCR/ABL* primers and probes that amplify *b3a2* and *b2a2* junctions have been reported [41, 42]. The primers and probes that amplify the *ABL* and *e1a2 BCR/ABL* junctions came from data reported by the Europe against Cancer Program [43, 44]. Normalized levels of the *BCR/ABL* ratios in the qRT-PCR assays were evaluated by comparisons to the *ABL* levels [10, 41, 42].

For a histopathological analysis of leukemic infiltration, femur, liver, spleen, and kidney tissues of recipients were fixed with 4% paraformaldehyde for 1 h, dehydrated with 70% ethanol, and embedded in paraffin, followed by the preparation of 5-µm sections. Hematoxylin–eosin (HE) staining was performed on each tissue section. Immunohistochemistry (IHC) with rabbit anti-hCD19 (Abcam, Cambridge, MA, USA) was performed after a graded alcohol dehydration and antigen retrieval using heated citrate buffer. Sections were studied by light microscopy (Axiovert 200; Carl Zeiss, Jena, Germany).

To understand how the different treatments affected the molecular pathways, western blot analyses using anti-human antibodies to phosphorylated CrkL and JAK2 were performed on the BM cells harvested from the different groups of recipient mice. The antibodies were obtained from Cell Signaling Technologies (Danvers, MA, USA).

### Statistical analyses

Statistical analyses were performed using the χ^2^ test for categorical variables and the Mann–Whitney U test for continuous variables. Analyses were performed using the SPSS 22.0 (IBM, Armonk, NY, USA) and GraphPad Prism 6.0 software packages (GraphPad Software, La Jolla, CA, USA), and differences with *P* < 0.05 were considered statistically significant.

## Results

### Overview of the RNA-Seq data

Approximately 62–76 million clean reads were obtained to establish four RNA-Seq libraries. As shown in Table 2, high percentages of reads (83.6–84.6%) were mapped. A total of 77.0–91.3% of the mapped reads were located within exons, whereas less than 19.6% of the mapped reads were located within introns and intergenic regions. These data indicated that we established four libraries with high quality, which allowed us to compare the transcriptomes between the LPCs and other cell fractions from the de novo Ph^+^ALL patients.

**Table 2:** RNA sequencing results of mRNA from the LPCs and other cells fractions in patients with de novo Ph^+^ALL

Sample ID^a^	Clean reads, M^b^	Mapping rate, %^c^	Exons, %	Intron, %	Intergenic, %
LPC_1_	60	83.6	81.1	16.1	2.8
LPC_2_	56	84.4	77.0	19.6	3.4
Other Cell_1_	68	84.6	91.3	7.3	1.5
Other Cell_2_	74	83.8	87.7	10.4	1.9

^a^LPC_1_, LPC_2_ and Other Cell_1_, Other Cell_2_ are replicate from the LPCs and other cell groups

^b^Indicates millions of reads

^c^Uses the Sus scrofa 10.2 as the reference genome annotation to classify the mapping tags into the different regions. Ratio was calculated by the number of tags on each region divided by the total tags on the whole genome

### *JAK2* mRNA and phospho-JAK2 were more highly expressed in the LPCs fraction than in the other cell fractions in patients with de novo Ph^+^ALL

**Fig. 2:**
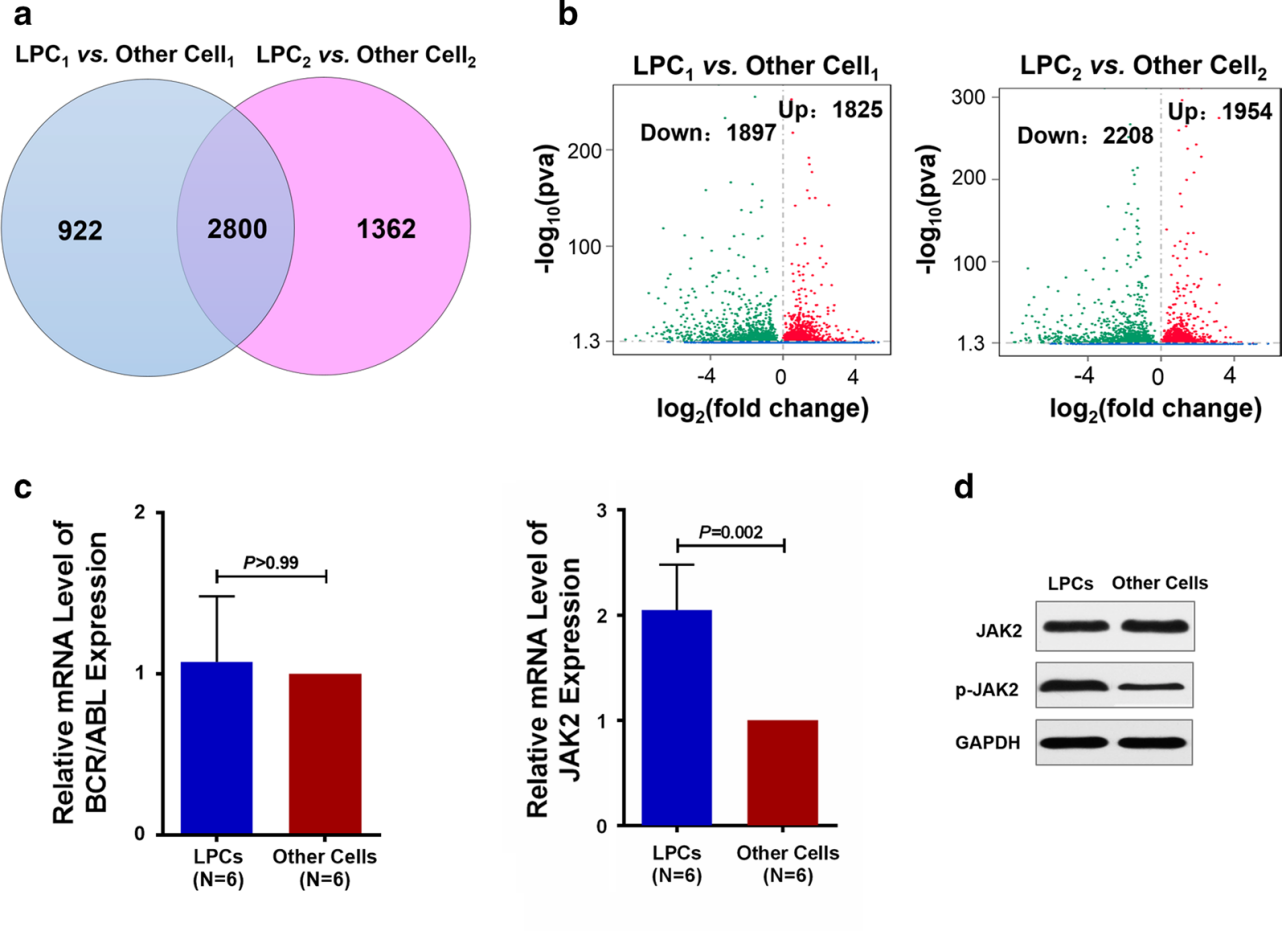
Comparative results of gene expression levels and the distribution of genes differentially expressed between the sorted LPCs and other cells fractions in patients with de novo Ph^+^ALL. **a** Venn diagram showing the genes detected by RNA-Seq that were differentially expressed between the LPC_1_ and Other Cell_1_ fractions (*light blue circle*), between the LPC_2_ and Other Cell_2_ fractions (*light red circle*), and gene expression profiles common to both groups (*intersection*). **b** Scatter plot of differentially expressed genes (*left panel*, LPC_1_ vs. Other Cell_1_; *right panel*, LPC_2_ vs. Other Cell_2_). *Red points* represent up-regulated genes with fold changes >1.5 and P*adj* < 0.05. *Green points* represent down-regulated genes with fold changes <−1.5 and P*adj* < 0.05. *Blue points* represent genes that were not significantly different. **c** The relative mRNA expression levels of *BCR/ABL* and *JAK2* between the LPCs and other cells fractions from the patients with de novo Ph^+^ALL (N = 6) were analyzed using qRT-PCR assays. **d** Representative western blots of phospho-JAK2 and GAPDH were shown in the LPCs and other cells fractions sorted from the patients with de novo Ph^+^ALL

A Venn diagram shows that 3722 genes were differentially expressed between the LPC_1_ and Other Cell_1_ fractions sorted from patient No. 1 with de novo Ph^+^ALL, whereas 4162 genes were differentially expressed between the LPC_2_ and Other Cell_2_ fractions from patient No. 2, and 2800 differentially expressed genes were co-expressed in both patients (Fig. 2a). A scatter plot shows the differentially expressed genes (Fig. 2b left panel, LPC_1_ vs. Other Cell_1_; Fig. 2b right panel, LPC_2_ vs. Other Cell_2_) with |fold change| >1.5 and P*adj* < 0.05. There were 3722 genes differentially expressed between the LPC_1_ and Other Cell_1_ fractions. Among them, 1825 genes were up-regulated and 1897 genes were down-regulated. Moreover, 1954 genes were up-regulated and 2208 genes were down-regulated in the LPC_2_ fraction compared with their expression levels in the Other Cell_2_ fraction. Based on the RNA-Seq data from patients with de novo Ph^+^ALL, *JAK2* was differentially expressed and was more highly expressed in the LPC_1_ fraction than in the Other Cell_1_ fraction (1.60-fold change, *P* = 0.03) and was more highly expressed in the LPC_2_ fraction than the Other Cell_2_ fraction (1.79-fold change, *P* = 0.004).

The relative mRNA expression levels of *BCR/ABL* and *JAK2* between the LPCs and other cell fractions from the patients with de novo Ph^+^ALL (N = 6) were further analyzed using qRT-PCR assays. No significant difference was found in the *BCR/ABL* mRNA levels between the LPCs and other cell fractions from the patients with de novo Ph^+^ALL as measured by qRT-PCR (Fig. 2c, 1.07 ± 0.17-fold, *P* > 0.99). Consistent with the RNA-Seq results, *JAK2* mRNA levels in the LPCs fraction were significantly higher than those in the other cell fractions (Fig. 2c, 2.05 ± 0.19-fold, *P* = 0.002). Likewise, the significantly higher levels of JAK2 mRNA detected by qRT-PCR were further validated in the protein level of phospho-JAK2 in the LPCs fraction when compared to the other cell fractions by western blot (Fig. 2d).

### Cotreatment with nilotinib and ruxolitinib simultaneously induces an increase in apoptosis of Ph^+^ALL LPCs in vitro

**Fig. 3:**
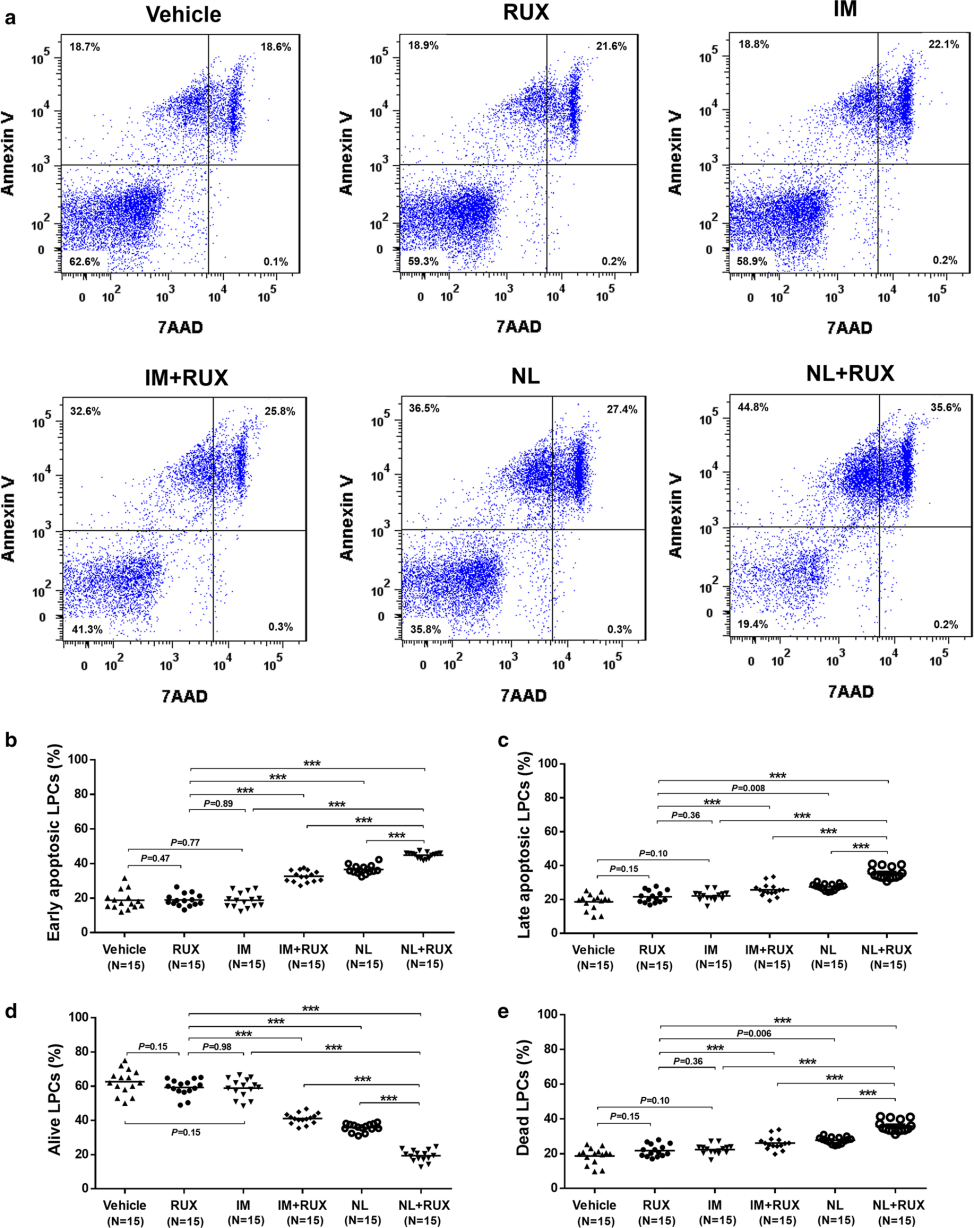
Effects of in vitro treatment with a single drug or different combinations of imatinib (IM), nilotinib (NL) and ruxolitinib (RUX) on LPCs from patients with de novo Ph^+^ALL. **a** Sorted LPCs from Ph^+^ALL patients (N = 5) were treated with DMSO vehicle, IM (5 µM), NL (5 µM) or RUX (300 nM) or their combination and cultured. Representative flow cytometric analysis of apoptotic LPCs was determined after Annexin-V/7-AAD staining following 48 h of treatment in each arm. **b** Percentages of the early apoptosis (Annexin-V^+^/7-AAD^−^) LPCs, late apoptosis (Annexin-V^+^/7-AAD^+^) LPCs (**c**), alive (Annexin-V^−^/7-AAD^−^) LPCs (**d**), and dead (7-AAD^+^) LPCs (**e**) were measured following Annexin-V/7-AAD staining after 48 h of treatment in each arm. Cultures were assayed in triplicate. All data from the independent experiments are presented as the mean ± SEM. Significance values ***P < 0.0001

To investigate anti-LPCs effects in vitro, the levels of drug-induced cell apoptosis were analyzed in the different treatment groups. As shown in Fig. 3, imatinib or ruxolitinib alone had no significant anti-LPCs effect in vitro compared with the effect in the vehicle-treated control group. Among the different treatment groups, cotreatment with nilotinib and ruxolitinib induced significantly higher levels of early apoptosis (Fig. 3b) and late apoptosis (Fig. 3c) in LPCs than did the other treatment groups. Alternatively, similar high levels of alive LPCs were found in the ruxolitinib-treated group (59.3 ± 1.2% vs. 62.6 ± 1.9%, *P* = 0.15) or imatinib-treated group (58.9 ± 1.4% vs. 62.6 ± 1.9%, *P* = 0.15) compared with the vehicle group. No significant difference was found between ruxolitinib-treated group and imatinib-treated group (59.3 ± 1.2% vs. 58.9 ± 1.4%, *P* = 0.98). In contrast, nilotinib (35.8 ± 0.6% vs. 59.3 ± 1.2%, *P* < 0.0001), imatinib combined with ruxolitinib (41.1 ± 0.8% vs. 59.3 ± 1.2%, *P* < 0.0001), or nilotinib combined with ruxolitinib (19.4 ± 0.9% vs. 59.3 ± 1.2%, *P* < 0.0001) resulted in significant reduction in alive LPCs compared with those in the ruxolitinib-treated group. Among these treatment groups, the levels of alive LPCs in the nilotinib combined with ruxolitinib-cotreated group exhibited the most marked reduction (Fig. 3d).

### Establishment of human Ph^+^ALL LPCs in the anti-CD122-conditioned NOD/SCID mouse xenograft assay

**Fig. 4:**
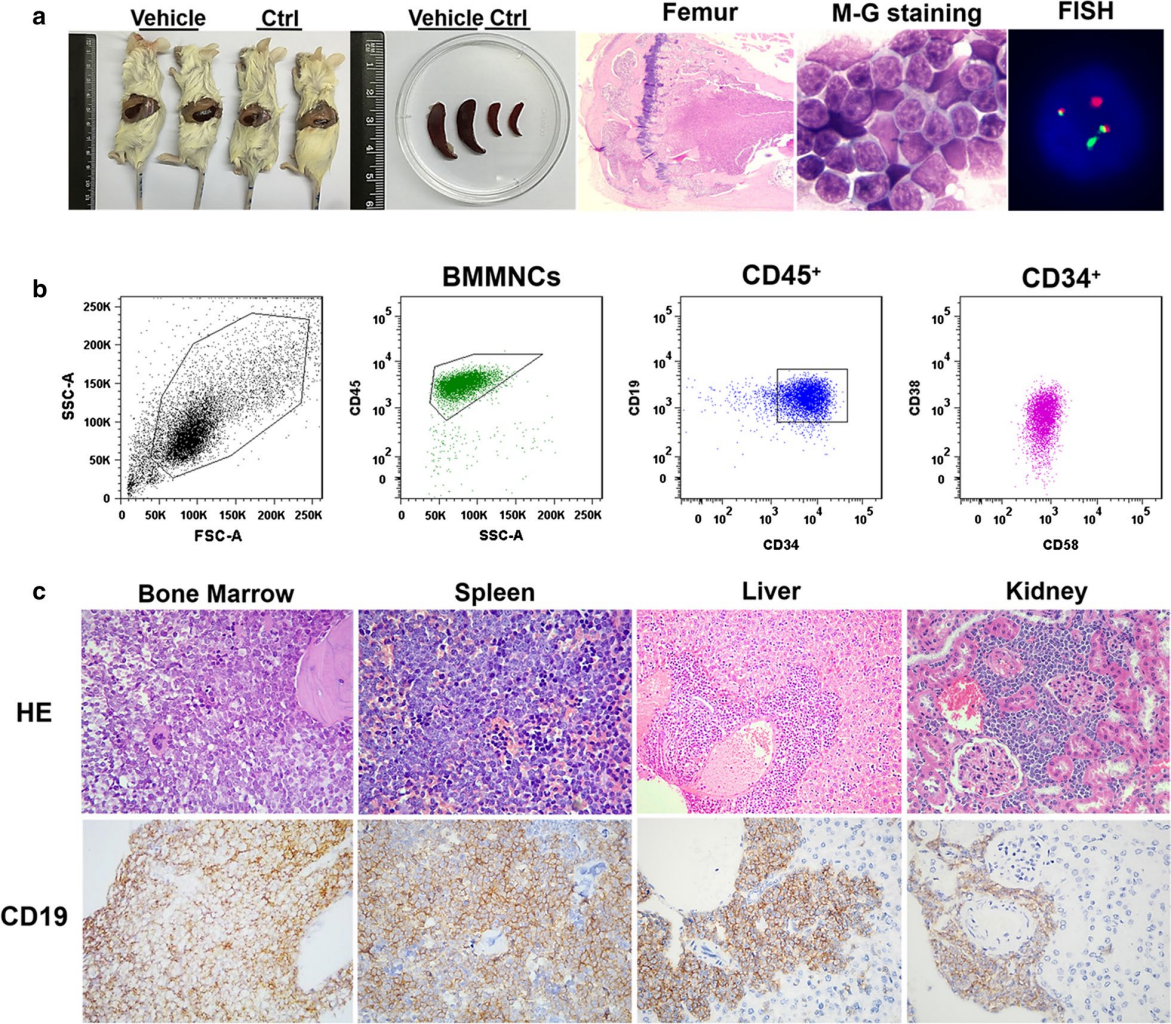
Establishment of a humanized Ph^+^ALL xenotransplant model using anti-CD122-conditioned NOD/SCID mice and intra-bone marrow injection (IBMI) with LPCs from Ph^+^ALL patients. **a** Compared with an irradiated non-transplanted control mouse (Ctrl), the mouse transplanted with LPCs and treated with vehicle (Vehicle) exhibited significant splenomegaly at 12 weeks post-transplant. A low-magnification image of human Ph^+^ALL engraftment in a bone section (*left panel*), May-Giemsa staining (*middle panel*) and fluorescence in situ hybridization (FISH) analysis (*right panel*) of leukemic blasts in the BM of the recipients (Vehicle). **b** Flow cytometric analysis demonstrated that the BMs of the recipients were efficiently engrafted with human Ph^+^ALL cells with an aberrant phenotype similar to that in the donor Ph^+^ALL patients. **c** Human engraftment of the BM, spleen, liver and kidney of the recipient (Vehicle) was further confirmed by HE staining (*upper panels*) and IHC with anti-hCD19 antibody (*lower panels*)

When the recipient mice exhibited ruffled fur and lethargy, the engraftment levels of human Ph^+^ALL were analyzed in the BM and spleen. Two months after the transplantation of 1 × 10^4^ LPCs and treatment with vehicle, the recipient mice exhibited splenomegaly. The engrafted human cells were further confirmed by morphologic and cytogenetic analyses (Fig. 4a). Flow cytometry analysis demonstrated that the BMs and spleens of the recipients were efficiently engrafted with human Ph^+^ALL cells with an aberrant phenotype similar to that in the donor Ph^+^ALL patients (Fig. 4b). There was widely disseminated disease, including significant leukemic infiltration into the BM, liver, spleen, and kidneys of the recipient mice, as shown by HE staining and IHC with anti-hCD19 (Fig. 4c). These results indicated that a humanized Ph^+^ALL xenotransplant model was successfully established using anti-CD122-conditioned NOD/SCID mice and IBMI with LPCs from Ph^+^ALL patients.

### The combination of nilotinib and ruxolitinib simultaneously exerts superior anti-LPCs activity in Ph^+^ALL than the other treatments

**Fig. 5:**
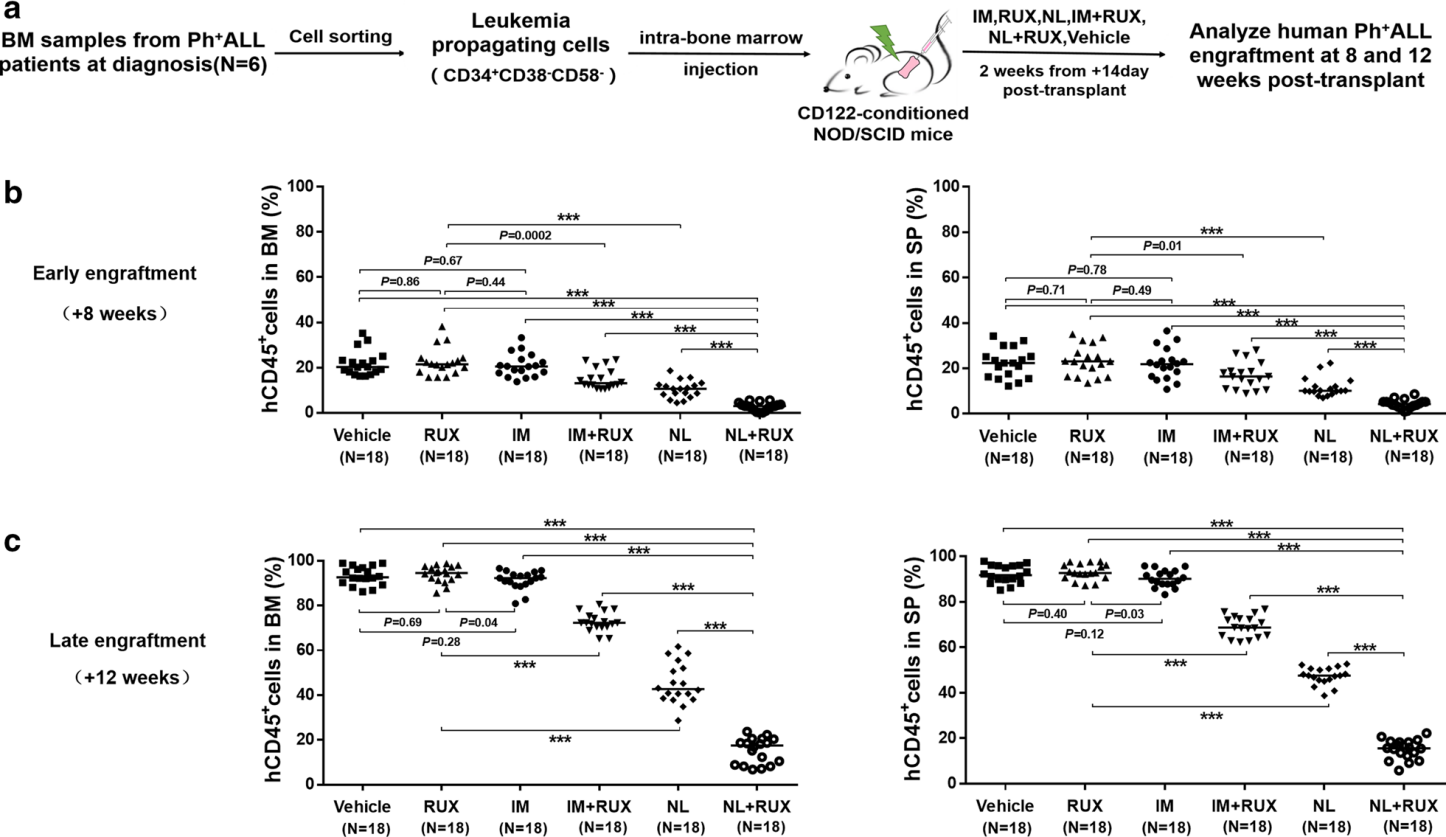
Effects of single drug treatment or different combinations of IM, NL and RUX on the engraftment of human Ph^+^ALL cells in the anti-CD122-conditioned NOD/SCID recipients transplanted with LPCs from Ph^+^ALL patients. **a** Experimental design for the in vivo experiments. LPCs sorted from newly diagnosed Ph^+^ALL patients (N = 6) were transplanted by IBMI into 5-week-old, sub-lethally irradiated (2.1 Gy of total body irradiation from a ^60^Co source) and anti-CD122-conditioned NOD/SCID mice. From +14 days post-transplantation, the recipient mice were randomly administered with vehicle (10% NMP-90% PEG 300), IM (100 mg/kg/day), NL (75 mg/kg/day), RUX (30 mg/kg/day), IM (100 mg/kg/day) combined with RUX (30 mg/kg/day), or NL (75 mg/kg/day) combined with RUX (30 mg/kg/day) via oral gavage for 14 days. **b** The engraftment levels of human Ph^+^ALL CD45^+^ cells in the BMs and spleens of mice under different treatment conditions were analyzed by flow cytometry at 8 weeks (N = 6 patients, N = 3 mice per patient per treatment group) and **c** at 12 weeks post-transplant (N = 6 patients, N = 3 mice per patient per treatment group) or until the mice were moribund

**Fig. 6:**
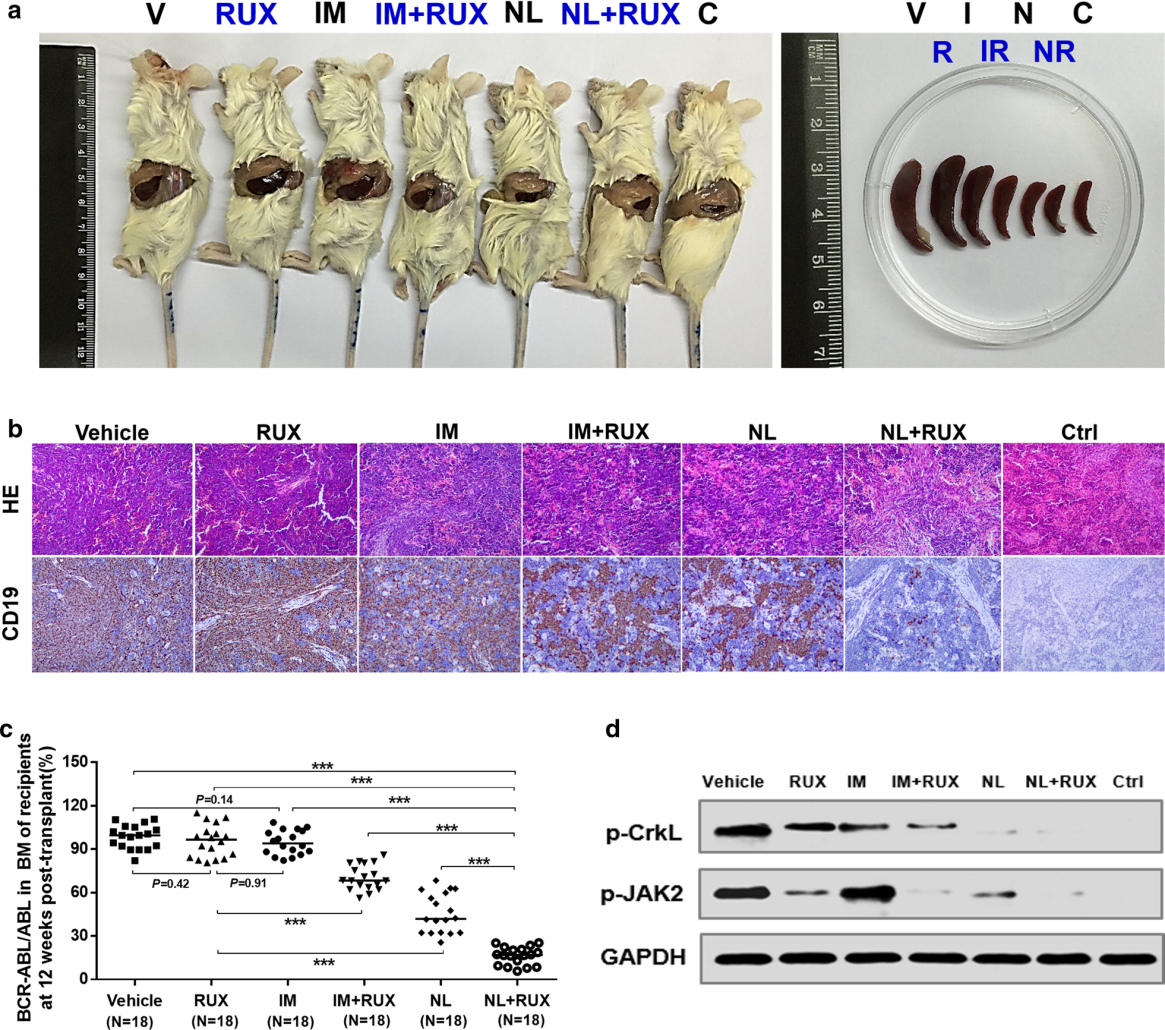
Cotreatment with NL and RUX exhibited the most effective anti-LPC effect. LPCs sorted from newly diagnosed Ph^+^ALL patients (N = 6) were transplanted by IBMI into 5-week-old, sub-lethally irradiated (2.1 Gy of total body irradiation from a ^60^Co source) and anti-CD122-conditioned NOD/SCID mice. From +14 days post-transplantation, the recipient mice were randomly administered with vehicle (10% NMP-90% PEG 300), IM (100 mg/kg/day), NL (75 mg/kg/day), RUX (30 mg/kg/day), IM (100 mg/kg/day) combined with RUX (30 mg/kg/day), or NL (75 mg/kg/day) combined with RUX (30 mg/kg/day) via oral gavage for 14 days. **a** Representative images of splenomegaly in the mice under different treatment conditions and a control NOD/SCID mouse (Ctrl) without receiving the Ph^+^ALL LPCs transplantation. **b** Differences in human engraftment were further confirmed by HE staining (*upper panels*) and IHC with anti-hCD19 antibody labeling (*lower panels*) of the spleens in the recipient mice treated with the different drugs and the Ctrl mice. **c** The engraftment analysis of *BCR/ABL*-expressing BM cells using a TaqMan-based qRT-PCR assay in the recipient mice at 12 weeks post-transplant. **d** Representative western blots of phospho-CrkL, phospho-JAK2, and GAPDH in the bone marrow cells of humanized mice transplanted with Ph^+^ALL LPCs following treatment with vehicle, single agents or a combination of RUX and IM or NL mice, and Ctrl mice. All data from the independent experiments are presented as the mean ± SEM. Significance values: ***P < 0.0001

To further evaluate the effects of selective BCR–ABL and/or JAK2 inhibition therapy on Ph^+^ALL LPCs in vivo, the recipient mice transplanted with LPCs were randomized to different treatment groups on day 15 post-transplant and were treated with various single or dual drug interventions for 14 days (Fig. 5a). At 8 weeks (Fig. 5b) and 12 weeks (Fig. 5c) post-transplant, similar high levels of human Ph^+^ALL engraftment were observed in the imatinib-treated mice and the vehicle-treated control mice. Consistent with the anti-LPCs effects in vitro, ruxolitinib alone had no significant anti-LPCs effect in the recipient mice, but this drug reduced Ph^+^ALL engraftment more significantly when ruxolitinib was administered with imatinib or nilotinib. Notably, treatment with the combination of nilotinib and ruxolitinib, compared with imatinib, nilotinib, or ruxolitinib treatment alone or imatinib combined with ruxolitinib, led to the most significant reduction in human Ph^+^ALL engraftment in the BMs and spleens of the recipients (Fig. 5b, c). Moreover, HE staining and IHC with anti-hCD19 demonstrated that the infiltrating levels of the transplanted LPCs were significantly lower in the spleen tissues of the nilotinib and ruxolitinib-cotreated mice than in those of mice in the other treatment groups (Fig. 6b). Further evidence that the most effective anti-LPCs effect occurred with the combination treatment was derived by the engraftment analysis of *BCR/ABL* expressing cells using a TaqMan-based qRT-PCR assay (Fig. 6c).

### Cotreatment with nilotinib and ruxolitinib is more effective in reducing phospho-JAK2 and phospho-CrkL activity in vivo

To determine the activities of JAK2 in humanized mice with Ph^+^ALL LPCs following treatment with single agents or a combination of ruxolitinib and imatinib or nilotinib, levels of phospho-JAK2 were examined by western blot analysis (Fig. 6d). Among the different treatment options, the combination of nilotinib and ruxolitinib was the most effective at reducing phospho-JAK2 levels in the recipients, whereas imatinib did not reduce the level of phospho-JAK2.

The phosphorylation levels of CrkL (Fig. 6d), the kinase substrate of BCR–ABL, were evaluated. We found that nilotinib or nilotinib treatment combined with ruxolitinib inhibited BCR–ABL kinase activity completely, whereas the phospho-CrkL levels did not change significantly when the recipient mice were treated with ruxolitinib alone.

These results are consistent with the engraftment levels evaluated using flow cytometry (Fig. 5b, c), histopathological analyses (Fig. 6b), and qRT-PCR assays (Fig. 6c), indicating that the combination of nilotinib and ruxolitinib more effectively reduced the LPCs capacity in immunodeficient mice through a deeper suppression of JAK2 activity than either single agent or the combination of imatinib and ruxolitinib. However, further comparison of the total JAK and CrkL protein is required to illuminate whether the change in phosphorylation was due to reduced amounts of total protein.

## Discussion

Using RNA-Seq and qRT-PCR, the current study revealed that JAK2 was more highly expressed in the sorted LPCs than in the other cell fractions in patients with de novo Ph^+^ALL. Furthermore, we provided pre-clinical evidence that combination treatment with a selective JAK1/JAK2 inhibitor (ruxolitinib) and nilotinib more effectively eradicated imatinib-insensitive primary LPCs than either ruxolitinib or TKIs alone by reducing the activities of phospho-CrKL and phospho-JAK2. These data indicate that simultaneously inhibiting BCR–ABL and JAK2 activities in LPCs is more effective than using single agents for patients with de novo Ph^+^ALL.

For decades, Ph^+^ALL has been regarded as the ALL subgroup with the worst outcome [2, 3]. The current management of Ph^+^ALL patients relies on the use of a TKI with or without chemotherapy followed by allo-HSCT. However, relapse remains the critical obstacle of treatment failure even after allo-HSCT. Therefore, the identification of alternative approaches to effectively prevent relapse in Ph^+^ALL patients is urgently needed.

JAK signaling pathways are required for cytokine and growth factor signaling [19–22]. The downstream molecules, which belong to the STAT family, are activated by JAKs. The JAK2–STAT5 pathway plays critical roles in normal hematopoiesis [19, 20] and CML leukemogenesis [21, 45, 46]. Recent studies have suggested that the JAK2–STAT5 pathway may provide putative survival signals to CML stem/progenitor cells; thus, the addition of ruxolitinib to a TKI therapy improved the therapeutic efficacy in CML both in vitro and in immunodeficient mice [26–28]. Therefore, therapeutically targeting the JAK2–STAT5 pathway appears to be a promising anti-LPCs management in CML [26–28].

Nilotinib is a second-generation TKI with a potent binding affinity for BCR–ABL tyrosine kinase [47, 48]. The advantages of nilotinib include its high in vitro affinity for BCR–ABL tyrosine kinase, and the improved molecular remission in the treatment of chronic-phase CML with this drug [47, 48]. Moreover, the combination of nilotinib with high-dose cytotoxic drugs in induction treatment, which comprised concurrent vincristine, daunorubicin, and prednisolone, as well as either 5 courses of consolidation followed by 2-year maintenance with nilotinib, or allo-HSCT, achieved significantly higher cumulative complete molecular remission and 2-year hematologic relapse-free survival rates than imatinib for patients with Ph^+^ALL [2, 49, 50]. Moreover, nilotinib’s low incidence of adverse events enhances drug compliance, making nilotinib more attractive than imatinib in the treatment of Ph^+^ALL [49].

The anti-CD122-conditioned NOD/SCID xenograft mouse assay with IBMI and the finding that LPCs were enriched in the CD34^+^CD38^−^CD58^−^ fraction in Ph^+^ALL patients provided us with a useful LPCs target and xenograft model for examining the anti-LPCs efficacy of drug treatment in Ph^+^ALL [10]. Indeed, the in vivo oral administration of ruxolitinib and nilotinib for 2 weeks significantly reduced infiltrated human leukemic cells to a greater extent in multiple hematopoietic tissues than did nilotinib or imatinib monotherapy. In contrast, ruxolitinib had no anti-LPCs effects when administered alone and was unable to augment the immediate effects of TKIs in rapidly decreasing the leukemia burden during the initial phase of therapy. However, the nilotinib-mediated inhibition of the BCR–ABL kinase might restore the requirement for cytokine-dependent JAK signaling and sensitize the residual LPCs to cotreatment with ruxolitinib [28]. Therefore, effective nilotinib therapy sets a precondition in which ruxolitinib acquires therapeutic efficacy in maintaining Ph^+^ALL remission.

In line with the current study, Appelmann et al. [51]. reported that dasatinib-mediated inhibition of BCR–ABL kinase resensitizes residual leukemic B cells to JAK inhibition in a Ph^+^ALL mouse model. In the report by Appelmann et al., different candidate LPCs (polyclonal cytokine-independent LPCs were generated using the retroviral vector-mediated introduction of the p185^BCR−ABL^ isoform into BM progenitor cells derived from Arf^null^ C57BL/6 mice, followed by a 7-day expansion of the transduced progeny under B-cell-selective culture conditions) and a Ph^+^ALL mouse model (LPCs were intravenously injected into the tail veins of healthy, non-conditioned 8- to 10-week-old C57BL/6 mice to establish a Ph^+^ALL mouse model) were employed. Although the candidate LPCs, mouse models of Ph^+^ALL, and second-generation TKIs were utilized differently in the two studies, Appelmann et al. [51] and the current study provide consistent evidence for the effective anti-LPCs effect of the combination of ruxolitinib and a second-generation TKI in Ph^+^ALL treatment. Mallampati et al. [52] demonstrated that the inhibition of JAK activity in combination with BCR–ABL inhibition can effectively short-circuit mesenchymal stem cell-mediated TKI resistance, which provided a mechanistic rationale for the current and previous pre-clinical studies suggesting that cotreatment with a second-generation TKI and a JAK inhibitor may be more effective in eradicating LPCs in Ph^+^ALL.

The current study suggested that targeting cytokine signaling through the combination of ruxolitinib and nilotinib is a promising strategy to eradicate residual Ph^+^ALL LPCs in vitro and in vivo. We are aware, however, that further in depth analysis and functional validation of the RNA-seq data of the sorted LPCs and other cell fractions from patients with de novo Ph^+^ALL, as well as investigation of the modulation of the important apoptosis markers and cell cycle status during the different treatment are needed to explore the underlying mechanisms and to determine how these processes sensitize LPCs to the combination of nilotinib and ruxolitinib. Alternatively, it is conceivable that residual LPCs after nilotinib treatment can be rescued through a cytokine-triggered JAK2–STAT5 pathway, which requires further clarification.

## Conclusions

Albeit preliminary, the data presented here indicate that cotreatment with nilotinib and ruxolitinib can more effectively eliminate imatinib-insensitive LPCs through a deeper suppression of BCR–ABL/JAK2 activity both in vitro and in humanized Ph^+^ALL mice. Therefore, this pre-clinical study appears to provide a scientific rationale for using selective BCR–ABL/JAK2 dual inhibition as a promising anti-LPCs therapeutic approach for patients with de novo Ph^+^ALL.

## Abbreviations

Ph^+^ALL: Philadelphia chromosome-positive acute lymphoblastic leukemia
LPCs: leukemia-propagating cells
RNA-Seq: RNA sequencing
qRT-PCR: real-time quantitative polymerase chain reaction
JAK: Janus kinase
Ruxolitinib: JAK1/JAK2 inhibitor
BCR–ABL: breakpoint cluster region, Abelson
CML: chronic myeloid leukemia
TKIs: tyrosine kinase inhibitors
allo-HSCT: allogeneic hematopoietic stem cell transplantation
NOD/SCID: non-obese diabetic/severe combined immunodeficiency
STAT: signal transducer and activator of transcription
BM: bone marrow
B-ALL: B cell acute lymphoblastic leukemia
WHO: World Health Organization
BMMNCs: bone marrow mononuclear cells
MoAbs: monoclonal antibodies
Rh: recombinant human
IL-3: interleukin-3
Flt3-L: Flt3-ligand
SCF: stem cell factor
IL-2Rβ: interleukin-2 receptor β
IBMI: intra-bone marrow injection
HE: hematoxylin–eosin
IHC: immunohistochemistry
